# A Comprehensive Analysis of Phytochemical Composition, Acute Toxicity Assessment, and Antioxidant Potential of Ethanolic Extract of Carica Papaya Seeds

**DOI:** 10.7759/cureus.49686

**Published:** 2023-11-29

**Authors:** Sarikonda Sandhya Rani, T Vedavijaya, Karuna Sree P, Jaikumar Shanmugasundaram, C Deepalatha, M Ganga Raju, Suresh Babu Sayana

**Affiliations:** 1 Department of Pharmacology, Meenakshi Academy of Higher Education and Research, Hyderabad, IND; 2 Department of Pharmacology, Meenakshi Ammal Dental College and Hospital, Chennai, IND; 3 Department of Pharmacology, All India Institute of Medical Sciences, Guwahati, Guwahati, IND; 4 Department of Pharmacology, Panimalar Medical College Hospital and Research Institute, Chennai, IND; 5 Department of Pharmacology, Mamata Academy of Medical Sciences, Hyderabad, IND; 6 Department of Pharmacology, Gokaraju Rangaraju College of Pharmacy, Hyderabad, IND; 7 Department of Pharmacology, Government Medical College and General Hospital, Suryapet, IND

**Keywords:** health benefits, antioxidant activity, safety assessment, phytochemicals, carica papaya seeds

## Abstract

Background: Carica papaya seeds are rich in phytochemicals with potential health benefits, warranting safety and antioxidant assessments. This study comprehensively examined the ethanolic extract of Carica papaya seeds (EECPS) to elucidate its phytochemical composition, acute toxicity profile, and antioxidant activity.

Methods: Phytochemical analysis of EECPS revealed the presence of various bioactive compounds, including flavonoids, tannins, phenols, alkaloids, proteins, glycosides, and saponins. Additionally, the presence of sulfuric acid was confirmed. Acute toxicity assessment involved oral administration of EECPS at 2000mg/kg body weight to Wistar rats, with a 14-day observation period. General parameters, body weight changes, and histopathological examination of kidney and liver tissues were evaluated. Antioxidant activity was assessed using the 2,2-Diphenyl-1-picrylhydrazyl (DPPH) assay, and the half-maximal inhibitory concentration (IC50) value of EECPS was compared to that of gallic acid.

Results: Phytochemical analysis confirmed the diverse composition of EECPS, suggesting its potential health benefits and biological activity. Acute toxicity assessment revealed no adverse effects, with rats exhibiting normal behavior, weight stability, and no histopathological abnormalities in vital organs. The gallic acid IC50 value was determined to be 5.73±0.02 µg/mL, indicating its antioxidant potency. EECPS exhibited antioxidant properties in a dose-dependent manner, with higher concentrations demonstrating increased DPPH free radical quenching capacity. The IC50 value for EECPS was calculated from the dose-response curve to be 39.41±1.61 µg/mL (expressed as mean ± standard error of the mean (SEM)).

Conclusion: The phytochemical analysis of EECPS highlights its diverse composition and potential health benefits. Acute toxicity studies in rats confirm its safety for oral administration, with no adverse effects observed. EECPS exhibits significant antioxidant activity, as indicated by its IC50 value. These findings suggest that EECPS holds promise for therapeutic use and health applications. However, further research is needed to determine its precise antioxidant potential. Subchronic and chronic toxicity studies are recommended to establish its safety profile definitively and unlock its full potential for healthcare and nutrition.

## Introduction

Carica papaya, commonly known as papaya, is a tropical fruit celebrated for its delicious flavor and nutritional value [1]. Beyond its fruit, the seeds of Carica papaya have gained recognition for their potential health benefits attributed to their phytochemical composition. These seeds have a long history of traditional use in various cultures for their medicinal properties, including their role as natural remedies for digestive disorders and parasites [1,2].

The seeds of Carica papaya are a rich source of bioactive compounds, which have been the subject of increasing scientific interest [3]. Phytochemical analysis of these seeds has revealed the presence of diverse compounds, including flavonoids, tannins, phenols, alkaloids, proteins, glycosides, and saponins [4,5]. These compounds are known for their antioxidant, antimicrobial, anti-inflammatory, and anticancer properties [6,7]. The presence of these bioactive constituents suggests that Carica papaya seeds may offer a wide range of potential health benefits, making them a subject of interest for researchers and health enthusiasts [8,9].

One area of focus in the study of Carica papaya seeds is their antioxidant activity. Oxidative stress, caused by an imbalance between free radicals and antioxidants in the body, is associated with various chronic diseases, including cancer, cardiovascular diseases, and neurodegenerative disorders [9]. Antioxidants play a crucial role in neutralizing free radicals and protecting cells from oxidative damage. The antioxidant potential of Carica papaya seeds, as assessed by methods such as the 2,2-Diphenyl-1-picrylhydrazyl (DPPH) assay, has been investigated to understand their ability to combat oxidative stress and potentially reduce the risk of these diseases.

Furthermore, safety assessments are essential before the incorporation of any natural product into healthcare or dietary practices. Toxicity studies are critical to determine the safety profile of Carica papaya seed extracts. Acute toxicity studies in animal models provide valuable insights into the immediate effects of high-dose exposure. In this regard, the acute toxicity assessment of ethanolic extract of Carica papaya seeds (EECPS) is a fundamental step in evaluating its safety for human consumption or therapeutic use. This assessment involves monitoring general observations, body weight changes, and histopathological examination to ensure the absence of adverse effects.

Given the promising phytochemical composition and potential health benefits of Carica papaya seeds, it is important to conduct a comprehensive analysis that includes phytochemical characterization, acute toxicity evaluation, and antioxidant assessment. Such a study can provide valuable information for researchers, healthcare professionals, and consumers interested in harnessing the health-promoting properties of these seeds. Moreover, as the use of natural remedies and dietary supplements continues to gain popularity, a thorough understanding of the safety and efficacy of Carica papaya seeds becomes increasingly relevant.

This study aims to contribute to the existing body of knowledge by providing insights into the phytochemical composition, safety profile, and antioxidant activity of EECPS. The results of this research can help inform future studies, guide potential therapeutic applications, and promote the responsible use of Carica papaya seeds as a natural health supplement.

## Materials and methods

Study location and duration

This research was conducted at Mamata Academy of Medical Sciences, Hyderabad, India. The study spanned a six-month period, commencing in January 2022 and concluding in June 2022.

Collection and preparation of Carica papaya seeds

Seed Collection

Fresh Carica papaya seeds were systematically collected from mature fruits. These fruits are obtained from home gardens, ensuring the selection of viable and fully ripened seeds. The seeds of Carica papaya were authenticated by Dr. P. Suresh Babu, Assistant Professor, Department of Botany, Government Degree College, Kukatpally, Hyderabad, India.

Cleaning and De-pulping

The collected seeds underwent a meticulous cleaning process to eliminate any residual fruit pulp or extraneous materials. This step was essential to maintain the purity of the seeds for subsequent analyses.

Drying Process

To attain the desired level of moisture content, the cleaned seeds were uniformly spread out and subjected to controlled air-drying conditions. This drying process aims to achieve optimal dryness.

Powder Preparation

Once adequately dried, the seeds were finely ground into a powder using a laboratory grinder. This step facilitated the efficient extraction of bioactive compounds from the seeds.

Preparation of EECPS

For the preparation of the ethanolic extract using Soxhlet extraction, ethanol was deliberately selected as the solvent due to its exceptional ability to dissolve a diverse array of phytochemicals present in the powdered Carica papaya seeds. The extraction process was carried out employing a Soxhlet extractor, wherein the finely ground Carica papaya seed powder was enclosed within a thimble and loaded into the Soxhlet apparatus. Ethanol was heated, facilitating its evaporation and ascent through the seed powder within the thimble. It then condensed and dripped back into the flask, establishing a continuous and thorough extraction cycle. This meticulous process extended over several hours to ensure the maximal extraction of bioactive compounds. Subsequently, the extracted ethanol solution underwent concentration to eliminate the solvent, resulting in the acquisition of a crude ethanolic extract enriched with bioactive components sourced from the Carica papaya seeds. This ethanolic extract, obtained through Soxhlet extraction, served as the foundation for subsequent phytochemical analyses and experimental investigations.

Phytochemical analysis

Tests for Alkaloids (Dragendorff's Test)

Sample preparation: 2-3 ml of concentrated EECPS.

Reagent: Dragendorff's reagent (0.85 g bismuth subnitrate, 10 g potassium iodide in 10 ml acetic acid, and 40 ml water).

Test procedure: Add a few drops of Dragendorff's reagent to the sample.

Conditions: Room temperature, immediate observation for an orange-brown precipitate.

Tests for Tannins and Phenolic Compounds

Sample preparation: 2-3 ml of either aqueous or ethanolic EECPS.

Reagents: Lead acetate solution (saturated), dilute iodine solution (0.1% to 1%).

Test Procedures

For tannins: Add a few drops of lead acetate solution.

For phenolic compounds: Add a few drops of dilute iodine solution.

Conditions: Room temperature, observe for immediate color changes.

Tests for Flavonoids

Sample preparation: Use the residue from initial tests.

Reagents: Sulphuric acid (66% or 80%), sodium hydroxide (2 M).

Test Procedures

Sulphuric acid test: Add sulfuric acid and observe the color change.

Sodium hydroxide test: Incrementally add sodium hydroxide.

Conditions: Room temperature, immediate color observation.

Tests for Proteins (Biuret Test)

The presence of proteins in the seed extracts was assessed using the Biuret test.

Sample preparation: A small amount of EECPS, diluted if necessary.

Reagent: Biuret reagent (1.5 g copper sulfate, 6 g sodium potassium tartrate in 500 ml of 0.1 N sodium hydroxide).

Test procedure: Add Biuret reagent and look for a color change to violet.

Conditions: Room temperature, observe for color change within a few minutes.

*Tests for Glycosides* *(Keller-Killiani Test)*

Sample preparation: An aliquot of EECPS in a layered test setup.

Reagents: Glacial acetic acid, 1-2% ferric chloride, and concentrated sulfuric acid.

Test procedure: Add specific reagents and observe the formation of a reddish-brown color at the interface.

Conditions: Room temperature, the color change observed after a few minutes.

Tests for Saponins (Foam Test)

Sample preparation: Shake a small amount of EECPS or dry powder with water.

Reagent: Water.

Test procedure: Observe the formation and persistence of foam.

Conditions: Room temperature, stable foam observed after vigorous shaking for a few minutes.

Determination of Isoliquiritigenin Using Liquid Chromatography-Mass Spectrometry (LC-MS)

Aim: The primary aim of this phase was to ascertain the molecular mass of isoliquiritigenin within the EECPS.

Chemicals and reagents: The analysis was conducted using LC-MS grade formic acid, methanol, and water.

Instrumentation: High-resolution mass spectrometry was carried out using a Q-ToF G6540B instrument by Agilent Technologies, CA, USA, coupled with an Agilent 1200 infinity series Ultra-High-Performance Liquid Chromatography (UHPLC) system.

Data acquisition and processing: Data acquisition and subsequent processing were conducted using the MassHunter workstation (Agilent Technologies, CA, USA).

Assessing acute toxicity of EECPS

In the assessment of acute toxicity for the EECPS, the extract was orally administered to the test subjects at a specific dosage of 2000 mg per kilogram of body weight. This dosing regimen was chosen to evaluate the immediate effects and potential adverse reactions that may occur shortly after ingestion. The purpose of this assessment was to determine if the extract, when given at this relatively high dose, would cause any observable signs of toxicity, adverse symptoms, or harm to the test subjects. This acute toxicity evaluation is a crucial initial step in understanding the safety profile of EECPS before considering further studies or applications. The test subjects' responses and any observed effects were carefully monitored and recorded to assess the extract's safety at this dosage level.

DPPH assay for antioxidant potential

Chemicals and Instrumentation

The DPPH assay utilized DPPH, methanol, gallic acid, ethanol, distilled water, a 96-well plate, and a multimode reader.

Procedure

Sample preparation: The ethanolic extract was dissolved in ethanol to achieve a concentration of 1 mg/ml. Serial dilutions were then prepared from this stock solution.

Gallic acid solutions: Solutions of gallic acid at varying concentrations (ranging from 1 µg/ml to 20 µg/ml) were prepared using distilled water.

DPPH reagent solution: A 125 µM DPPH reagent solution was prepared in methanol.

Reaction setup: In 96-well plates, 50 µL of either the extract or gallic acid solutions were combined with 150 µL of the 125.

Ethical approval

Ethical approval for animal experimentation was duly obtained from the Institutional Animal Ethics Committee (IAEC) Mamata Academy of Medical Sciences, Hyderabad, India under approval number 01/MAMS/IAEC/2021.

Statistical analysis

Qualitative data in phytochemical analysis were detailed descriptively, indicating the various bioactive compounds present or absent. High-resolution mass spectrometry facilitated the identification of isoliquiritigenin by equating molecular masses. Observational data from acute toxicity and histopathological examinations were presented qualitatively. For the DPPH assay, the half-maximal inhibitory concentration (IC50) values for gallic acid and EECPS, denoted as Mean±SEM (standard error of the mean), were compared using a t-test or analysis of variance (ANOVA) to discern statistical significance. Regression analysis of percentage inhibition data elucidated dose-response relationships. Repeated measures confirmed the EECPS IC50 values' accuracy. All analyses were conducted using the Statistical Package for the Social Sciences (IBM SPSS Statistics for Windows, IBM Corp., Version 28.0, Armonk, NY), setting the significance threshold at p < 0.05, ensuring the study's analytical rigor.

## Results

Phytochemical composition analysis

Qualitative phytochemical analysis of the EECPS revealed the presence of various bioactive compounds.

Flavonoids

A positive response in the alkaline reagent test confirmed the presence of flavonoids.

Tannins

Tannins were detected through the formation of a precipitate using the lead acetate solution test.

Phenols

The presence of phenolic compounds was confirmed by a color change in the dilute iodine solution test.

Alkaloids

Alkaloids were identified by a positive result in Dragendorff's test, evident as a reddish-brown precipitate.

Proteins

A violet color formation in the Biuret test confirmed the presence of proteins.

Glycosides

The Keller-Killiani test yielded a red color upon hydrolysis, indicating the presence of glycosides.

Saponins

Positive results in the foam formation test indicated the presence of saponins.

Additionally, the presence of sulfuric acid was confirmed via a positive result in the sulfuric acid test. These findings collectively suggest the diverse phytochemical composition of EECPS, contributing to its potential health benefits and biological activity (Table 1).

**Table 1:** Qualitative phytochemical analysis of ethanolic extract of Carica papaya seeds (EECPS)

Phytochemicals	Tests	EECPS
Flavonoids	Sulphuric acid test, alkaline reagent test	Present
Tannins and Phenols	Lead acetate solution test, dilute iodine solution test	Present
Alkaloids	Dragendorff’s test	Present
Proteins	Biuret test	Present
Glycosides	Keller-Killiani test	Present
Saponins	Foam formation test	Present

Quantitative phytochemical analysis - Isoliquiritigenin in EECPS seed using LC-MS

**Figure 1:**
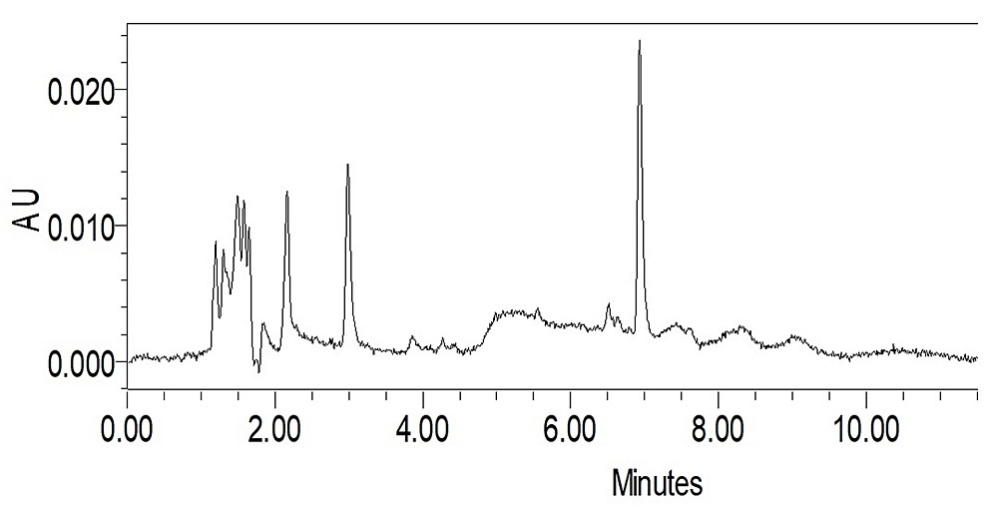
Quantitative phytochemical analysis: Isoliquiritigenin in ethanolic extract of Carica papaya seed using LC-MS LC-MS: Liquid chromatography-mass spectrometry

The analysis using LC-MS provided valuable insights into the molecular mass of isoliquiritigenin present in the EECPS. The specific molecular mass of isoliquiritigenin was determined, which is crucial for its identification and quantification (Figure 1).

Acute toxicity assessment

The acute toxicity assessment of EECPS (2000mg/kg body weight) in Wistar rats resulted in the following observations: No visible changes in skin, fur, eyes, or mucous membranes were observed during the 14-day observation period. Respiratory patterns remained normal, and there were no signs of distress or abnormal behavior. No tremors, convulsions, salivation, diarrhea, lethargy, sleep, or coma were observed in any of the rats. Individual rat weights remained stable throughout the 14-day period, indicating no significant weight loss or gain due to extract administration.

Histopathological examination

**Figure 2:**
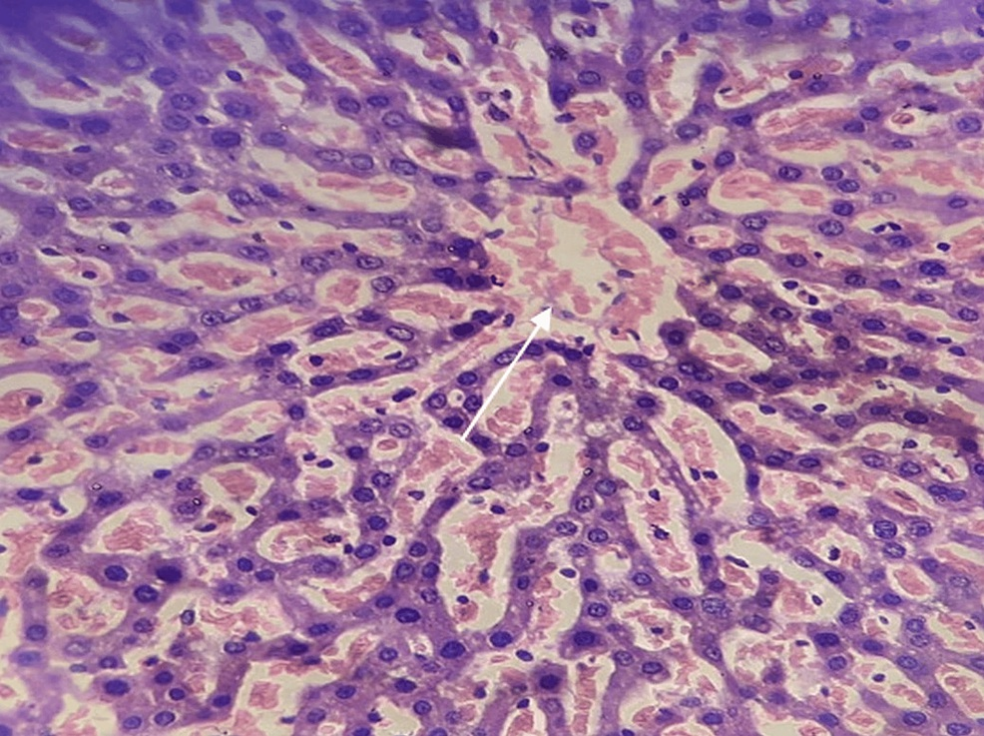
Histopathological evaluation of liver tissue post-acute toxicity assessment with ethanolic extract of Carica papaya seeds (EECPS) The arrow indicates in the figure the central vein of the liver.

**Figure 3:**
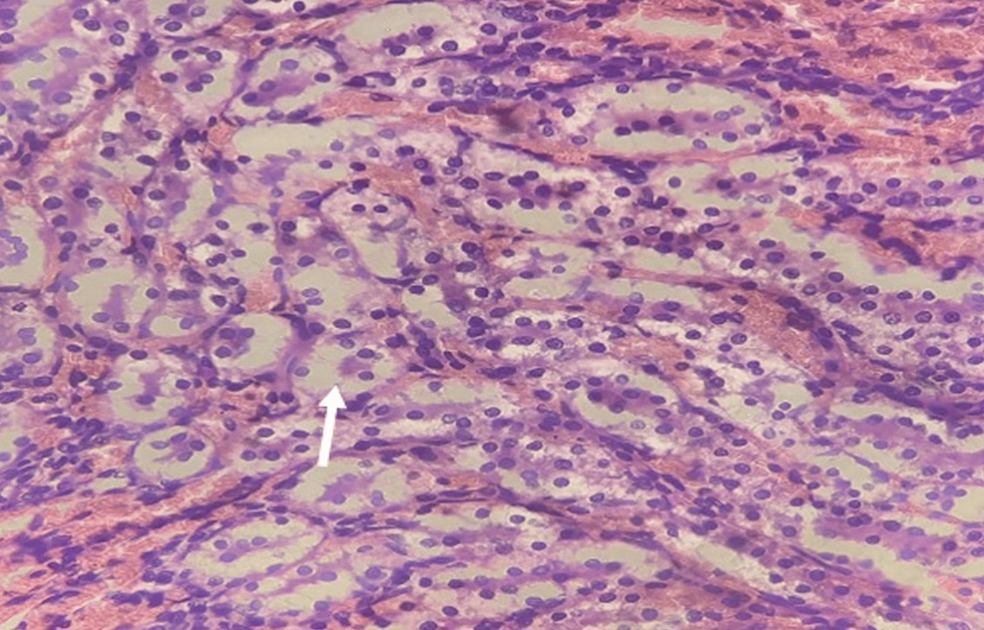
Histopathological evaluation of kidney tissue post-acute toxicity assessment with ethanolic extract of Carica papaya seeds (EECPS) The arrow indicates in the figure normal renal tubules.

Kidney and liver tissues were examined on the 14th day, revealing no significant abnormalities, lesions, or tissue damage associated with the administered dose of EECPS (2000mg/kg body weight). This suggests the safety of the tested dose within the scope of this acute toxicity study (Figures 2, 3).

Quantitative analysis of dose-dependent antioxidant efficacy of gallic acid via DPPH assay

Table 2 systematically shows the results of the DPPH radical scavenging assay, aiming to elucidate the antioxidant potential of gallic acid.

**Table 2:** Dose-dependent antioxidant activity of gallic acid: IC50 value and percentage inhibition in DPPH assay DPPH: 2,2-Diphenyl-1-picrylhydrazyl, IC50: Half-maximal inhibitory concentration

S. No	Concentration (μg/ml)	Percentage Inhibition (%)	p-value
1	1	15.06	p > 0.05
2	2	21.79	p < 0.05
3	4	35.34	p < 0.001
4	6	49.58	p < 0.001
5	8	63.97	p < 0.001
6	10	87.80	p < 0.001

The IC50 value, a critical determinant in antioxidant studies, for gallic acid stands at 5.73±0.02 µg/mL. This value signifies the concentration at which gallic acid neutralizes 50% of DPPH radicals. The SEM, represented as ±0.02 µg/mL, accentuates the consistency and precision of the measurements.

Detailing the data further

Initiating the assay at a concentration of 1 µg/mL, gallic acid demonstrates an inhibition of 15.06%, hinting at its inherent antioxidant strength. Elevating the concentration to 2 µg/mL results in an increased inhibition of 21.79%. A notable rise in antioxidant activity is observed at 4 µg/mL, with an inhibition percentage of 35.34%. Further increments in concentration to 6 µg/mL and 8 µg/mL yield inhibition percentages of 49.58% and 63.97%, respectively. The pinnacle of the assay, at 10 µg/mL, reveals gallic acid’s robust antioxidant prowess, inhibiting a substantial 87.80% of DPPH radicals (Table 2).

Graphical representation of gallic acid's dose-dependent DPPH scavenging efficacy

**Figure 4:**
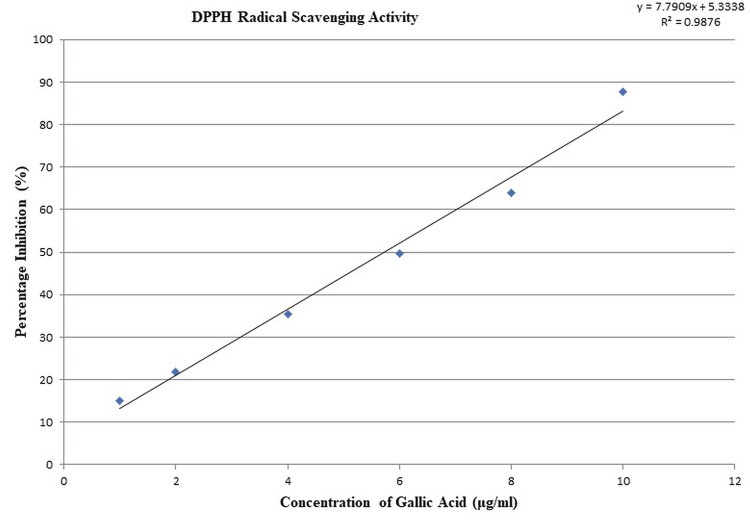
Analysis of concentration-dependent inhibition: Observing progressive antioxidant activity DPPH: 2,2-Diphenyl-1-picrylhydrazyl

Figure 4 provides a visual rendition of gallic acid's concentration-versus-inhibition relationship. The linear regression equation associated with the plotted data is:

y=7.7909x+5.3338

R2=0.9876

The high coefficient of determination (R² value of 0.9876) signifies an exceptional fit of the data to the linear model, implying a strong and direct correlation between gallic acid concentration and its antioxidant capacity. Intrinsically, as the concentration of gallic acid surges, a corresponding rise in its ability to neutralize DPPH radicals is depicted. The steep gradient, reflected in the slope of 7.7909, accentuates this potent dose-response relationship, confirming gallic acid's stature as an eminent antioxidant compound (Figure 4).

Antioxidant potential of the EECPS using DPPH scavenging assay

The results delineate the antioxidant efficacy of the EECPS as determined by the DPPH radical scavenging assay. A pivotal metric in this assessment is the IC50 value, a parameter that denotes the concentration requisite to neutralize 50% of DPPH radicals. For EECPS, the derived IC50 value stands at 39.41±1.61 µg/mL (expressed as Mean±SEM). It's pertinent to highlight that the accompanying ±1.61 µg/mL signifies the SEM, underscoring the precision and reproducibility of the obtained data.

Table 3 sequentially tabulates varying concentrations of EECPS alongside their concomitant DPPH inhibition percentages.

**Table 3:** Evaluation of the antioxidant activity of ethanolic extract of Carica papaya seeds (EECPS): A detailed DPPH assay analysis DPPH: 2,2-Diphenyl-1-picrylhydrazyl

S. No	Concentration (μg/ml)	Percentage Inhibition (%)	p-value
1	0.975	20.53	p < 0.01
2	1.95	25.30	p < 0.001
3	3.9	26.39	p < 0.001
4	7.81	32.02	p < 0.001
5	15.625	44.94	p < 0.001
6	31.25	50.88	p < 0.001

Commencing at a nominal concentration of 0.975 µg/mL, EECPS manifests a DPPH inhibition of 20.53%, an early indicator of its antioxidant prowess. Progressing along an approximately logarithmic concentration gradient, a concomitant escalation in the DPPH inhibition percentage is discernible. Illustratively, concentrations of 1.95 µg/mL and 3.9 µg/mL result in inhibitions of 25.30% and 26.39% respectively. A salient observation emerges at elevated concentrations. Specifically, at 125 µg/mL, EECPS exhibits an impressive DPPH inhibition of 90.48% (Table 3).

Concentration-dependent DPPH radical scavenging activity of EECPS

**Figure 5:**
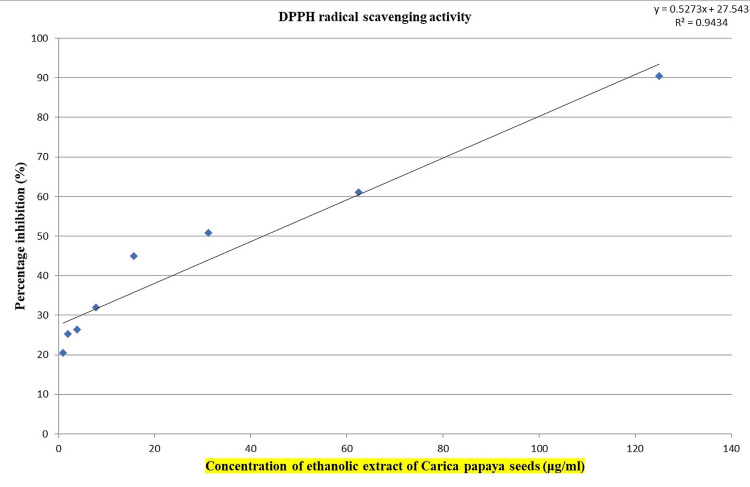
Concentration-dependent antioxidant activity of EECPS: Exploring the dose-response pattern of EECPS in DPPH assay DPPH: 2,2-Diphenyl-1-picrylhydrazyl, EECPS: Ethanolic extract of Carica papaya seeds

Figure 5 graphically represents the relationship between the concentration of the EECPS and its corresponding DPPH radical scavenging activity. The linear regression model derived from the data is represented by the equation:

y=0.5273x+27.543

R2=0.9434

The high coefficient of determination (R² = 0.9434) indicates a strong correlation between the variables. The upward trajectory of the graph, as implied by the positive slope (0.5273), signifies that increasing concentrations of EECPS correspond with enhanced antioxidant activity. Analyzing the results alongside the tabulated data, one can anticipate a pronounced incline in the graph around and beyond the IC50 value, marking heightened DPPH inhibition percentages (Figure 5).

## Discussion

The EECPS stands as a testament to the intricate blend of nature's offerings and their therapeutic potential. Our comprehensive analysis not only adds depth to the understanding of EECPS but also echoes the broader significance of phytochemical-rich extracts in health sciences.

Our study on EECPS revealed a plethora of bioactive compounds, a finding that aligns well with previous works (Mawa et al., 2013; Kong et al., 2021) [10,11]. Beyond the mere identification, the variety - spanning flavonoids, tannins, phenols, and others - is indicative of a dynamic biochemical matrix. These compounds, historically and scientifically, have been linked with a broad spectrum of therapeutic activities. Their established roles in antioxidant, anti-inflammatory, and anticancer mechanisms (Cushnie & Lamb, 2005; Meng et al., 2021; Farombi & Owoeye, 2011) [12-15] underscore the potential applicability of EECPS in various therapeutic interventions. This chemical diversity possibly sets EECPS as a potential reservoir for multi-targeted therapeutic avenues.

The precision of our methodology was illuminated with the detection of isoliquiritigenin through LC-MS. This flavonoid, previously identified in Carica papaya seeds (Mawa et al., 2013) [14], has garnered attention for its pronounced antioxidative and anti-inflammatory actions (Meng et al., 2021) [13]. The presence of such a potent compound raises pivotal questions: Could isoliquiritigenin be the primary player in EECPS's overall activity? How does it interact or synergize with other detected compounds? Delving into these queries could form the basis for subsequent research, unraveling the molecular intricacies of EECPS's effects.

A therapeutic agent's efficacy is best complemented by its safety profile. Our findings in Wistar rats provide preliminary but promising data on EECPS's safety, mirroring prior studies that report a favorable toxicological profile for Carica papaya seed extracts (Singh et al., 2020; Kamalakkannan & Prince, 2006) [16,17]. While the current data is reassuring, it also underscores the need for extended evaluations. Comprehensive studies spanning longer durations, varied doses, and different biological matrices would provide a holistic understanding of EECPS's safety spectrum.

Our comparative assessment with the renowned antioxidant, gallic acid, has further buttressed EECPS's radical-scavenging prowess. Despite gallic acid's superior IC50 value, the EECPS's performance, consistent with earlier findings (Kong et al., 2021) [11], hints at the collaborative actions of its diverse phytochemical constituents.

This study serves as a cogent piece in the ever-evolving puzzle of botanical extracts, emphasizing the need to bridge traditional knowledge with modern scientific methodologies. EECPS, with its myriad of bioactive compounds, beckons further exploration, both for therapeutic applications and molecular insights.

Limitations of the study include our investigation into the EECPS offered revealing insights, yet some limitations warrant attention. The seeds sourced were bound by geographical and seasonal specificity, potentially not encapsulating the global seed variability influenced by diverse climates and harvest times. The use of LC-MS, while precise, could overlook compounds present in trace amounts, and its outcomes might be influenced by calibration differences. Moreover, our toxicological assessment, confined to acute exposure in Wistar rats, offers a mere glimpse into EECPS's safety profile, possibly overlooking long-term effects and not necessarily reflecting responses in other species, including humans.

Furthermore, while gallic acid served as a reference for assessing antioxidant potential, a multi-referential approach might have offered a more comprehensive perspective. Emphasis on isoliquiritigenin did not fully explore its concentration relative to other compounds or its interactions with them. The study's focus on a specific EECPS dosage leaves the effects of alternate dosages uncharted. Although numerous bioactive compounds were identified, their specific therapeutic effects and underlying mechanisms remained untouched. The potential interplay of EECPS with other substances wasn't examined, raising questions about combined effects. Lastly, the study's methodologies and settings might restrict the general applicability of our findings, suggesting the need for diverse future investigations.

## Conclusions

Our study has revealed the diverse phytochemical composition of EECPS, including the presence of isoliquiritigenin. Importantly, our acute toxicity assessment has demonstrated the safety of EECPS at the tested dosage. Additionally, our findings indicate its significant antioxidant potential as evidenced by the DPPH assay. These results, derived from our study, highlight the promising health-related properties of EECPS. To unlock its full therapeutic potential, further investigations, including in vivo and clinical studies, are essential. Our research lays the groundwork for a more comprehensive exploration of the potential benefits of EECPS in various health-related applications.
